# The risk trajectory between preoperative fasting glucose and common digestive tract cancer-specific mortality in the FIESTA cohort involving 6865 Chinese patients

**DOI:** 10.7150/jca.31184

**Published:** 2019-08-07

**Authors:** Dan Hu, Feng Peng, Xiandong Lin, Hejun Zhang, Yan Xia, Jinxiu Lin, Xiongwei Zheng, Wenquan Niu

**Affiliations:** 1 Department of Pathology, Fujian Cancer Hospital & Fujian Medical University Cancer Hospital, Fuzhou, Fujian, China; 2 Department of Cardiology, The First Affiliated Hospital of Fujian Medical University, Fuzhou, Fujian, China; 3 Department of Radiobiology, Fujian Cancer Hospital & Fujian Medical University Cancer Hospital, Fuzhou, Fujian, China; 4 Institute of Clinical Medical Sciences, China-Japan Friendship Hospital, Beijing, China

**Keywords:** digestive tract cancer, fasting glucose, prognosis, mortality.

## Abstract

**Backgrounds:** High blood glucose or hyperglycemia is an established risk factor for the development and progression of cancer at many sites, whereas data on the relevance between low blood glucose or hypoglycemia and cancer survival are lacking.

**Aims:** We aimed to assess the shape of risk trajectory between preoperative fasting glucose and postoperative digestive cancer-specific mortality in Chinese.

**Methods:** In total, 6865 patients who underwent radical surgery for esophageal cancer (n=2535), gastric cancer (n=3012) and colorectal cancer (n=1318) during 2000-2010 were followed up as of December 2015. All patients received neither chemotherapy nor radiotherapy before and after the surgery. Optimal cutoff points were determined using survival tree analysis.

**Results:** The median follow-up time was 44.9 months (range: 0.5-188.9 months), with 1065 deaths from esophageal cancer, 1331 from gastric cancer and 412 from colorectal cancer. Using fasting glucose (4.36, 6.09] mmol/L as the reference group, hazard ratios for fasting glucose ≤4.36, (6.09, 8.95], (8.95, 11.5] and >11.5 mmol/L were 1.35 (95% confidence interval: 1.19, 1.54), 2.82 (2.57, 3.11), 3.56 (3.10, 4.08) and 4.27 (3.67, 4.97), respectively (*p*<0.001).

**Conclusions:** Our findings indicate a U-shaped risk trajectory between preoperative fasting glucose and digestive tract cancer-specific mortality in Chinese. Further external validation is warranted.

## Introduction

It is widely recognized that high blood glucose or hyperglycemia is an established risk factor for the development and progression of cancer 1-3. The underlying mechanisms connecting hyperglycemia and cancer have been long debated 4,5, and a possible explanation is that chronic hyperglycemia can mediate physiological alteration and metabolic distortion that further leads to organ dysfunction, infection, cancer progression and other pathophysiological consequences 6. We have recently in the Fujian prospective investigation of cancer (FIESTA) study, assessed the prognostic association of preoperative metabolic syndrome and its components with the disease-specific mortality of esophageal squamous cell carcinoma 7, gastric cancer 8 and colorectal cancer 9, respectively. Our findings consistently indicated that hyperglycemia was an independent significant predictor of poor prognosis for common digestive tract cancer. However, a systematic literature search has failed to reveal any proofs linking low blood glucose or hypoglycemia to cancer survival. An emerging body of evidence supports that hypoglycemia can induce a pro-inflammatory state 10, which has a detrimental role in carcinogenesis 11. We therefore developed a hypothesis that hypoglycemia may be associated with an increased mortality risk for digestive tract cancer. To test this hypothesis, in *post hoc* analysis of the FIESTA study, we aimed to assess the shape of risk trajectory between preoperative fasting glucose and postoperative specific mortality of three types of common digestive tract cancer mentioned above, overall and by cancer type.

## Methods

### The FIESTA study

The FIESTA study is an ongoing investigation of preoperative factors for predicting disease-specific mortality of common digestive tract cancer, including sites at esophagus, stomach and colon and rectum.

### Study patients

Using data from the FIESTA cohort 7-9,12-20, a total of 6865 eligible patients who underwent radical surgery for esophageal cancer (n=2535), gastric cancer (n=3012) and colorectal cancer (n=1318) at Fujian Provincial Cancer Hospital (the current Fujian Cancer Hospital & Fujian Medical University Cancer Hospital) and survived hospitalization between January 2000 and December 2010 were analyzed in the current study, and they were followed up as of December 2015. The FIESTA study got approval from the Ethical Committee of the Fujian Provincial Cancer Hospital, and all patients gave written informed consent.

Digestive tract cancer was confirmed with preoperative biopsy or postoperative pathologic examination. All patients are unrelated Han Chinese, and they received neither chemotherapy nor radiotherapy before and after the surgery.

### Follow-up evaluation

Follow-up was conducted annually after discharge either at the Out-Patient Department of Fujian Provincial Cancer Hospital or through calling or sending post letters in case of no-show on scheduled time. If death occurred during follow-up, the exact date was traced either through his/her relatives or medical reports. The study design, recruitment procedure, eligibility criteria and follow-up assessment have been described previously 7-9,13,14,16.

### Baseline data

At enrollment, each patient was asked to complete a self-designed structured questionnaire covering information on social demographic and anthropometric characteristics, including age, gender, cigarette smoking status, alcohol drinking status and family cancer history. Meanwhile, body weight and height were measured to calculate body mass index (BMI). Blood pressure was also measured, and hypertension was defined as systolic blood pressure ≥140 mm Hg or diastolic blood pressure ≥90 mm Hg or intake of antihypertension agents.

### Clinicopathologic data

A pair of cancer tissue and near normal tissue was cut from each patient during radical resection. All tissue samples were formalin-fixed and paraffin-embedded, and they were pathologically analyzed at the Department of Pathology, Fujian Provincial Cancer Hospital.

Clinicopathologic data were got from medical charts and pathological reports, including invasion depth, regional lymph node metastasis, distant metastasis, differentiation, tumor embolus and tumor nodes metastasis (TNM) stage (I, II, III and IV) 21.

Fasting (at least 8 hours) venous blood sample was collected into the EDTA-K2 anticoagulative tubes at the morning of undergoing the surgery. Plasma triglycerides, total cholesterol, and high-density lipoprotein cholesterol and low-density lipoprotein cholesterol were measured per standard procedures. Fasting blood glucose was determined by an automated glucose oxidase method.

### Survival time definition

Cancer-specific survival time was defined as the time from the date of radical surgery to the date of the death from specific types of three common digestive tract cancer under study or the date of the last follow-up, whichever occurred first.

### Statistical analysis

The optimal cutoff points of preoperative fasting glucose concentrations were determined in survival tree analysis implemented by the STREE program (http://c2s2.yale.edu/software/stree/). In detail, only preoperative fasting glucose was incorporated in the analysis, along with clinical endpoint and survival time, and node value was extracted to constitute a partition of study patients.

Difference in survival rates was presented in Kaplan-Meier curve and judged by Log-rank test. Risk prediction, expressed as hazards ratio (HR) and 95% confidence interval (95% CI), was estimated using the Weibull hazards regression model. Statistical analysis was implemented using the Stata/SE software (StataCorp, TX, version 14.1). Study power was estimated using the PS-Power Simple Size software (version 3.1.2).

## Results

The median follow-up time was 44.9 months (range: 0.5-188.9 months), with 2808 deaths from digestive tract cancer (1065 from esophageal cancer, 1331 from gastric cancer and 412 from colorectal cancer). The overall median survival time was 113.4 months.

Among all study patients, four potential cutoff points of fasting glucose concentrations preoperatively at 4.36, 6.09, 8.95, 11.5 mmol/L were determined in survival tree analysis. Accordingly, 6865 patients were classified into five groups according to above glucose thresholds in mmol/L: fasting glucose concentrations ≤4.36 (group I: n=891), (4.36, 6.09] (group II: n=3469), (6.09, 8.95] (group III: n=1440), (8.95, 11.5] (group IV: n=430) and >11.5 (group V: n=635).

Baseline demographic and clinicopathologic characteristics stratified by five fasting glucose groups are shown in Table 1.

**Figure 1:**
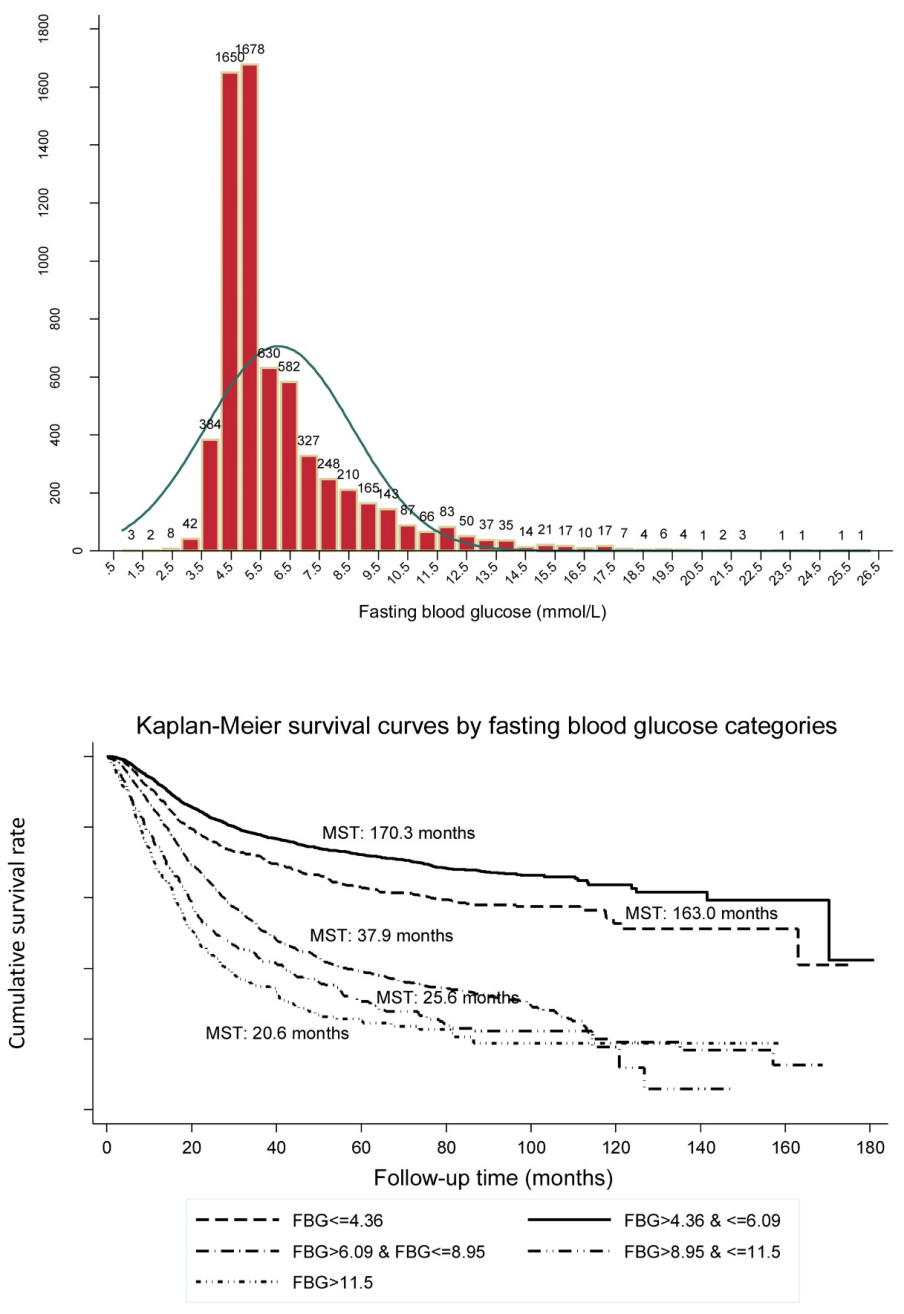
The distribution of preoperative fasting blood glucose in all patients with common digestive tract cancer (the upper panel) and Kaplan-Meier survival curves according to preoperative fasting blood glucose (the lower panel).

The distributions of preoperative fasting glucose concentrations in 0.5 mmol/L increments among all study patients are presented as a frequency histogram (Figure 1, the upper panel).

The Kaplan-Meier curve showed good discrimination of cancer-specific survival for all five fasting glucose groups (Log-rank test *p*<0.001), as shown in Figure 1 (the lower panel). It is worth noting that patients in group II (median survival time [MST]: 170.3 months) had the best prognosis, followed by group I (MST: 163.0 months), group III (MST: 37.9 months), group IV (MST: 25.6 months) and group V (MST: 20.6 months).

Using group II as the reference group, HRs for group I, group III, group IV and group V were 1.35 (95% CI: 1.19, 1.54), 2.82 (95% CI: 2.57, 3.11), 3.56 (95% CI: 3.10, 4.08) and 4.27 (95% CI: 3.67, 4.97), respectively (all *p*<0.001) after adjusting for confounding factors in overall analysis (Table 2). The power to detect statistical significance was over 99% for above estimates.

Significance persisted after grouping patients by cancer type and TNM stage. Risk prediction was particularly evident for patients with gastric cancer, and for patients at early stages (I and II).

## Discussion

This large prospective cohort study of patients with common digestive tract cancer provides strong evidence for a U-shaped risk trajectory between preoperative fasting glucose and postoperative digestive tract cancer-specific mortality in Chinese with a median follow-up of 44.9 months. Compared with patients with preoperative fasting glucose concentrations ranging 4.36-6.09 mmol/L, the cancer-specific mortality risk was significantly increased for both lower (than 4.36 mml/L) and higher (than 6.09 mmol/L) fasting glucose. The relation was particularly evident for gastric cancer and early stages.

The observations that elevated blood glucose is associated with high risk or poor prognosis of cancer at many sites have been widely made in the medical literature 22-25. However, relevant data on low blood glucose or hypoglycemia are sparse and only restricted to cancer risk 26. As a symptom of cancer, hypoglycemia is drawing much concern from a biological aspect. For example, low glucose stress can decrease cellular NADH and mitochondrial ATP in colonic epithelial cancer cells 27. Additionally, low glucose can enhance the cytotoxicity of metformin to cancer cells both in vitro and in vivo 28. We in the FIESTA cohort study show that preoperative fasting glucose concentration less than 4.36 mmol/L was independently associated with a 35% increased mortality risk of common digestive tract cancer, especially for early stages (58% increase) and gastric cancer (47% increase). Irrespective of underlying mechanisms, low fasting glucose before the surgery can clearly identify patients with poorer postoperative prognosis who could benefit from closer monitoring.

In this study, we employed survival tree analysis to determine optimal cutoff points for fasting glucose. A conventional method to determine cutoffs is the adoption of receiver operating characteristic (ROC) curve, yet this method often produces different points across studies, which limits the generalizability. For this case, several splitting criteria have been developed, including classification and regression trees (CART) and multivariate adaptive regression splines (MARS) 29,30. Although the relative merits of these criteria are not clearly resolved, survival tree-based method has been applicable to more general situations on the basis of scientific judgement 31.

Despite the clear strengths of the current study, including prospective design, large sample size and long follow-up interval, our findings should be interpreted within the context of the following limitations. This is a mono-center study, limiting the generalizability of findings, although it facilitates consistency of evaluation. Due to the difficulty in identifying an external group, we are unable to validate our findings in an independent population. In addition, data on drug regimens and medical treatment are not available, which might introduce a systematic bias and residual confounding. Importantly, only fasting glucose was measured before the surgery, and it is of great interest to monitor postoperative glucose, and if possible glycosylated hemoglobin, which is accurate and stable, to see its dynamic changes in predicting survival of digestive tract cancer. All resectable patients were recruited between January 2000 and December 2010, and during this period, remarkable advances in surgical techniques might introduce a possible bias. Findings were exclusively enrolled from a southern city in China, calling for external replications in other domestic or ethnic populations.

In conclusion, our findings provide strong evidence for a U-shaped risk trajectory between preoperative fasting glucose and digestive tract cancer-specific mortality in Chinese. This study highlights the importance of measuring fasting glucose for patients who undergo radical surgery for digestive tract cancer to inform risk assessment and identify patients in need of closer monitoring postoperatively. For practical reasons, we hope that this study will not remain just another end point of research but instead a beginning to establish background data to unveil the underlying mechanisms of glucose abnormalities in carcinogenesis.

## Figures and Tables

**Table 1:** Baseline characteristics of study patients with common digestive tract cancer according to preoperative fasting glucose

Characteristics	Preoperative fasting glucose (mmol/L)				
	Group I (≤ 4.36)	Group II (4.36, 6.09)	Group III (6.09, 8.95)	Group IV (8.95, 11.5)	Group V> 11.5
No. of patients	891	3469	1440	430	635
Age at cancer diagnosis (years)	55.48 (11.42)	56.84 (10.67)	58.79 (10.73)	59.66 (10.77)	59.88 (10.61)
Males	676 (75.87%)	2495 (71.92%)	1035 (71.88%)	300 (69.77%)	430 (67.72%)
Females	215 (24.13%)	974 (28.08%)	405 (28.13%)	130 (30.23%)	205 (32.28%)
Cigarette smokers	258 (29.52%)	881 (26.02%)	364 (25.60%)	108 (25.78%)	90 (14.98%)
Alcohol drinkers	118 (13.50%)	351 (10.38%)	142 (9.98%)	35 (8.35%)	38 (6.33%)
Family cancer history	93 (10.68%)	362 (10.71%)	151 (10.62%)	42 (10.02%)	34 (5.67%)
BMI (kg/m^2^)	22.62 (2.70)	22.56 (2.93)	23.25 (3.27)	23.54 (3.41)	22.90 (3.15)
BMI category					
≤ 24 kg/m^2^	726 (81.48%)	2418 (69.70%)	867 (60.21%)	239 (55.58%)	327 (51.50%)
(24, 28] kg/m^2^	148 (16.61%)	904 (26.06%)	467 (32.43%)	144 (33.49%)	184 (28.98%)
> 28 kg/m^2^	17 (1.91%)	147 (4.24%)	106 (7.36%)	47 (10.93%)	124 (19.53%)
SBP (mmHg)	120.34 (18.59)	122.85 (17.96)	128.61 (19.60)	129.89 (21.03)	131.45 (20.24)
DBP (mmHg)	75.67 (10.96)	76.75 (10.49)	79.17 (11.10)	79.61 (12.34)	79.94 (12.31)
TG (mmol/L)	1.04 (0.70)	1.14 (0.80)	1.35 (1.03)	1.50 (1.14)	1.48 (1.25)
TC (mmol/L)	4.36 (0.94)	4.68 (1.02)	4.72 (1.19)	4.63 (1.16)	4.64 (1.19)
HDLC (mmol/L)	1.12 (0.36)	1.15 (0.39)	0.98 (0.39)	0.94 (0.52)	0.92 (0.39)
LDLC (mmol/L)	2.91 (0.84)	3.06 (0.91)	3.11 (1.06)	3.01 (0.97)	3.02 (1.01)
Glucose (mmol/L)	4.03 (0.37)	5.02 (0.42)	7.18 (0.83)	9.97 (0.68)	14.11 (2.59)
Hypertension	181 (20.41%)	766 (22.16%)	526 (36.55%)	157 (37.12%)	128 (20.65%)
Cancer site					
Esophageal cancer	353 (39.62%)	1350 (38.92%)	530 (36.81%)	161 (37.44%)	141 (22.20%)
Gastric cancer	360 (40.40%)	1408 (40.59%)	684 (47.50%)	193 (44.88%)	367 (57.80%)
Colorectal cancer	178 (19.98%)	711 (20.50%)	226 (15.69%)	76 (17.67%)	127 (20.00%)
Invasion depth					
T1	69 (7.74%)	393 (11.33%)	102 (7.08%)	36 (8.37%)	43 (6.77%)
T2	116 (13.02%)	517 (14.90%)	169 (11.74%)	43 (10.00%)	105 (16.54%)
T3	514 (57.69%)	1829 (52.73%)	788 (54.72%)	221 (51.40%)	268 (42.20%)
T4	192 (21.55%)	730 (21.04%)	381 (26.46%)	130 (30.23%)	219 (34.49%)
Regional LNM					
N0	335 (37.60%)	1449 (41.77%)	431 (29.93%)	137 (31.86%)	161 (25.35%)
N1	280 (31.43%)	1005 (28.97%)	416 (28.89%)	111 (25.81%)	165 (25.98%)
N2	225 (25.25%)	821 (23.67%)	449 (31.18%)	129 (30.00%)	176 (27.72%)
N3	51 (5.72%)	194 (5.59%)	144 (10.00%)	53 (12.33%)	133 (20.94%)
Distant metastasis					
Negative	652 (73.18%)	2673 (77.05%)	997 (69.24%)	267 (62.09%)	451 (71.02%)
Positive	239 (26.82%)	796 (22.95%)	443 (30.76%)	163 (37.91%)	184 (28.98%)
Differentiation					
High	82 (9.20%)	273 (7.87%)	82 (5.69%)	27 (6.28%)	93 (14.65%)
Moderate	484 (54.32%)	1992 (57.42%)	720 (50.00%)	227 (52.79%)	282 (44.41%)
Low	325 (36.48%)	1204 (34.71%)	638 (44.31%)	176 (40.93%)	260 (40.94%)
Tumor embolus					
Negative	703 (78.90%)	2677 (77.17%)	982 (68.19%)	291 (67.67%)	124 (19.53%)
Positive	188 (21.10%)	792 (22.83%)	458 (31.81%)	139 (32.33%)	511 (80.47%)
TNM stage					
I	98 (11.00%)	513 (14.79%)	126 (8.75%)	38 (8.84%)	12 (1.89%)
II	230 (25.81%)	970 (27.96%)	323 (22.43%)	88 (20.47%)	171 (26.93%)
III	503 (56.45%)	1803 (51.97%)	813 (56.46%)	244 (56.74%)	339 (53.39%)
IV	60 (6.73%)	183 (5.28%)	178 (12.36%)	60 (13.95%)	113 (17.80%)

Abbreviations: BMI, body mass index; SBP, systolic blood pressure; DBP, diastolic blood pressure; TG, triglyceride; TC, total cholesterol; HDLC, high-density lipoprotein cholesterol; LDLC, low-density lipoprotein cholesterol; LNM, lymph node metastasis; TNM, tumor nodes metastasis. Data are expressed as either mean (standard deviation) or count (percentage).

**Table 2:** Risk prediction of preoperative fasting blood glucose in categories for common digestive tract cancer-specific mortality

Group	Adjustment*	Preoperative fasting glucose (mmol/L)				
		Group I (≤ 4.36)	Group II (4.36, 6.09]	Group III (6.09, 8.95]	Group IV (8.95, 11.5]	Group V > 11.5
Overall	Unadjusted	1.39 (1.23, 1.57) <0.001	Reference	2.78 (2.54, 3.05) <0.001	3.60 (3.16, 4.11) <0.001	4.28 (3.70, 4.95) <0.001
	Adjusted	1.35 (1.19, 1.54) <0.001	Reference	2.82 (2.57, 3.11) <0.001	3.56 (3.10, 4.08) <0.001	4.27 (3.67, 4.97) <0.001
Cancer site						
Esophageal cancer	Unadjusted	1.28 (1.07, 1.82) <0.001	Reference	1.80 (1.55, 2.10) <0.001	2.38 (1.92, 2.97) <0.001	2.49 (1.94, 3.18) <0.001
	Adjusted	1.26 (1.05, 1.51) 0.015	Reference	1.81 (1.56, 2.11) <0.001	2.40 (19.2, 3.01) <0.001	2.51 (1.95, 3.22) <0.001
Gastric cancer	Unadjusted	1.50 (1.23, 1.82) <0.001	Reference	3.30 (2.89, 3.78) <0.001	4.33 (3.58, 5.23) <0.001	5.10 (4.12, 6.31) <0.001
	Adjusted	1.47 (1.20, 1.81) <0.001	Reference	3.39 (2.93, 3.93) <0.001	4.18 (3.39, 5.14) <0.001	5.10 (4.05, 6.43) <0.001
Colorectal cancer	Unadjusted	1.44 (1.02, 2.04) 0.039	Reference	4.63 (3.62, 5.91) <0.001	5.84 (4.17, 8.18) <0.001	9.80 (6.87, 13.97) <0.001
	Adjusted	1.40 (1.05, 2.10) 0.026	Reference	5.00 (3.89, 6.42) <0.001	6.01 (4.25, 8.52) <0.001	9.99 (6.96, 14.32) <0.001
Stage						
I-II	Unadjusted	1.63 (1.20, 2.20) 0.002	Reference	4.09 (3.27, 5.11) <0.001	4.74 (3.41, 6.60) <0.001	5.99 (4.04, 8.87) <0.001
	Adjusted	1.58 (1.15, 2.16) 0.004	Reference	4.08 (3.23, 5.14) <0.001	4.89 (3.49, 6.87) <0.001	6.00 (4.01, 8.97) <0.001
III-IV	Unadjusted	1.26 (1.10, 1.44) 0.001	Reference	2.29 (2.07, 2.53) <0.001	2.97 (2.57, 3.43) <0.001	3.33 (2.85, 3.90) <0.001
	Adjusted	1.26 (1.09, 1.45) 0.001	Reference	2.32 (2.08, 2.57) <0.001	2.90 (2.49, 3.38) <0.001	3.29 (2.79, 3.88) <0.001

Data are expressed as hazard ratio (95% confidence interval) p value. *Adjusted variables included age, sex, body mass index, smoking, drinking, family cancer history, hypertension, triglyceride and total cholesterol.
